# The Effect of Nutrition Education on Intradialytic Weight Gain in Haemodialysis Patients: Systematic Review and Meta‐Analysis

**DOI:** 10.1002/nop2.70284

**Published:** 2025-08-22

**Authors:** Sevda Tüzün Özdemir, Zekiye Karaçam, Öznur Usta Yeşilbalkan

**Affiliations:** ^1^ Izmir Kavram Vocational School, Dialiysis Programme Izmir Turkey; ^2^ Department of Midwifery Faculty of Health Sciences, Aydın Adnan Menderes University Aydın Turkey; ^3^ Department of Internal Medicine Nursing, Faculty of Nursing, Ege University Izmir Turkey

**Keywords:** education, haemodialysis, intradialytic weight gain, meta‐analysis, nurse, systematic review

## Abstract

**Aim:**

This systematic review and meta‐analysis aimed to determine the impact of nutrition education on intradialytic weight gain (IDWG) in haemodialysis (HD) patients based on primary studies.

**Design:**

Systematic review and meta‐analysis.

**Methods:**

Ten databases were searched for randomised controlled trials between November 1st and November 30th, 2022. A total of 20 studies met the inclusion criteria and were analysed.

**Results:**

The meta‐analysis of 15 studies examining the effects of nutrition education on IDWG showed a statistically significant difference between the experimental and control groups (*p* < 0.0001). Subgroup analysis based on the duration of data collection indicated that this significant effect persisted in the 1st and 2nd months, as well as the 3rd and 4th months, but was not significant in the 5th and 6th months (*p* = 0.15).

**No Patient or Public Contribution:**

No patient or public contribution was used.

## 1 Introduction

In haemodialysis (HD) patients, the weight gained between two dialysis sessions is referred to as the intradialytic weight gain (IDWG) (Hanifi et al. 2019; Naseri‐Salahshour et al. 2020; Kurt et al. 2012). Excessive IDWG can lead to hypervolemia, particularly in patients with systemic illness, resulting in systemic disorders and complications, sometimes leading to premature termination of dialysis treatment (Goto et al. 2021). Premature completion of dialysis treatment in successive sessions exacerbates hypervolemia and dialysis inadequacy, thereby reducing the patient's quality of life. Moreover, high IDWG levels are associated with increased mortality rates due to hypertension, left ventricular hypertrophy, pulmonary oedema, congestive heart failure, and cardiac issues (Goto et al. 2021; Oller et al. 2018; Liu et al. 2020). For an ideal IDWG, it is recommended to have a weight gain of approximately 3%–5% of dry weight or 1–2 kg (Sentürk 2021). A study reported that 85% of HD patients had an IDWG exceeding 1.5 kg, with higher IDWG levels observed in young, male, and diabetic patients, which was associated with increased cardiovascular disease‐related mortality rates (Kalantar‐Zadeh et al. 2009). Similar findings have been reported in numerous other studies (Oller et al. 2018; Hara et al. 2020; Hecking and Wabel 2020).

It is reported that approximately 90% of HD patients do not adhere to the recommended diet therapy, and 52% do not adhere to IDWG recommendations (Günalay et al. 2017; Karabulutlu and Yılmaz 2019). Therefore, high adherence to the recommended diet programme is crucial in reducing systemic disorders and treatment‐related issues caused by high IDWG, improving quality of life and reducing mortality rates in HD patients (Kulaksız 2019; Naseri‐Salahshour et al. 2020; Beerendrakumar et al. 2018). Nutrition education provided to HD patients in the literature is reported to be effective in promoting desired dietary behaviours and reducing IDWG (Naseri‐Salahshour et al. 2020; Oshvandi et al. 2013; Balım and Pakyüz 2016; Kurt et al. 2012).

Haemodialysis nurses play a significant role in providing care for dialysis patients throughout the entire medical treatment process, meeting their needs and planning and implementing education programmes according to these needs (Tüzün and Akyol 2021; Ebrahimi et al. 2016; Liu et al. 2022, Liu et al. 2020). Planned education programmes, especially those involving interventions to control weight gain between HD sessions, are crucial for increasing adherence to diet programmes, improving the quality of life of HD patients, and alleviating treatment‐related symptoms (Ebrahimi et al. 2016; Aghakhani et al. 2012).

Although many experimental and quasi‐experimental studies have reported non‐adherence to IDWG in HD patients and the positive impact of nutrition education on this issue (Goto et al. 2021; Oller et al. 2018; Kalantar‐Zadeh et al. 2009; Hara et al. 2020; Naseri‐Salahshour et al. 2020; Oshvandi et al. 2013; Balım and Pakyüz 2016); These studies are further supported by comprehensive systematic reviews and meta‐analyses. Therefore, this research aims to address this gap by conducting a systematic review and meta‐analysis. This systematic review and meta‐analysis aim to determine the effect of nutrition education on IDWG in HD patients based on primary studies. The findings from this study are expected to contribute to reducing high mortality rates in HD patients by increasing adherence to IDWG and improving overall quality of life.

## 2 Methods

### 2.1 Establishment of the Problem

The studies eligible for this systematic review were selected according to the following criteria (PICOS): Population (P): HD patients. Intervention (I): Structured nutrition education. Comparison (C): No structured nutrition education or pre‐test results. Outcomes (O): Primary outcome: Intradialytic weight gain. Secondary outcomes: Systolic and diastolic blood pressure, quality of life, self‐efficacy, potassium, sodium, phosphorus, albumin, urea, creatinine and dialysis adequacy. Study design (S): Experimental and quasi‐experimental studies conducted in 2010 and later, published in English and Turkish.

### 2.2 Evidence Sources and Retrieval Strategies

Reviews for this systematic review and meta‐analysis study were conducted between the 1st and 30th November 2022. While reviews performed to access international publications included (“Dialysis AND Education”) AND (“intradialytic weight” OR “weight gain”) keywords and PubMed, Cochrane Library, EBSCO, Embase, Web of Science, PsycINFO, Cochrane, Turkish clinics, TR Index and Council of Higher Education‐National Thesis Center databases. For further reviews, reference lists of studies included in this study and previous studies on this issue were checked.

### 2.3 Study Inclusion and Exclusion Criteria

The inclusion criteria are as follows: (i) studies whose main objective was to investigate the effect of structured educational interventions on intradialytic weight gain in haemodialysis patients; (ii) the study population consisted of haemodialysis patients; (iii) outcome indicators included intradialytic weight gain, systolic and diastolic blood pressure, quality of life, self‐efficacy, potassium, sodium, phosphorus, albumin, urea, creatinine and dialysis adequacy; (iv) articles published in Turkish and English languages in 2010 or later; (v) included article types were randomised controlled trials and quasi‐experimental studies. Study exclusion criteria were as follows: (i) published in languages other than Turkish and English; (ii) studies with unclear research methods; (iii) full text not available; (iv) observational or animal experiments; (v) studies using nutrition/diet education outside HD patients.

### 2.4 Data Extraction

A data extraction tool, developed by the researchers, was utilised to collect data in this study. The tool was designed to capture information such as author and publication year, study location and timeframe, study design, sample size, group characteristics, type and method of training, comparison group and findings related to primary and secondary outcomes. The primary outcomes of interest included IDWG levels after nutrition education, while secondary outcomes encompassed systolic and diastolic blood pressure, quality of life, self‐efficacy, potassium, sodium, phosphorus, albumin, urea, creatinine and dialysis adequacy.

### 2.5 Literature Quality Evaluation

#### 2.5.1 Literature Quality Evaluation Process

This systematic review and meta‐analysis was carried out under the statement of Preferred Reporting Items for Systematic Reviews (PRISMA) (Page et al. 2021). The study protocol was registered in the PROSPERO database (ID: CRD42022349091) to prevent duplication of research, facilitate comparisons with studies in the planning stage, and reduce the risk of bias.

Throughout the study, a rigorous process was employed to minimise potential bias risk. This process included literature searches, selection of articles, data extraction and assessment of article quality. These tasks were independently conducted by two researchers and cross‐verified by a third researcher.

#### 2.5.2 Literature Quality Evaluation Method

The methodological quality of the included articles was assessed using the RoB 2 tool for randomised controlled trials and the ROBINS‐I tool for non‐randomised studies, both of which were developed by Cochrane (Sterne et al. 2019; Sterne et al. 2016).

### 2.6 Statistical Analysis

Data analyzes were performed using Review Manager 5.4.1 (The Nordic Cochrane Center, Copenhagen, Denmark), Comprehensive Meta‐Analysis Version 4 for meta‐regression and publication bias (Borenstein et al. 2022). Heterogeneity between studies was assessed using *χ* ^2^ test and *I* ^2^ statistic (Deeks et al. 2021). In this study, random‐effects model was used to incorporate heterogeneity among studies included in the meta‐analysis. The variables in the study were continuous variables, and the “Mean Difference (MD)” was calculated. A *p* value less than 0.05 in two‐tailed tests was considered statistically significant.

Sensitivity analysis was conducted through meta‐regression to assess the effects of outcome measurement time, study design, training programme, country and risk of bias on the primary outcome variable, IDWG. Subgroup analysis was performed for significant moderators identified through the meta‐regression analysis.

## 3 Results

### 3.1 Search Studies

**FIGURE 1:**
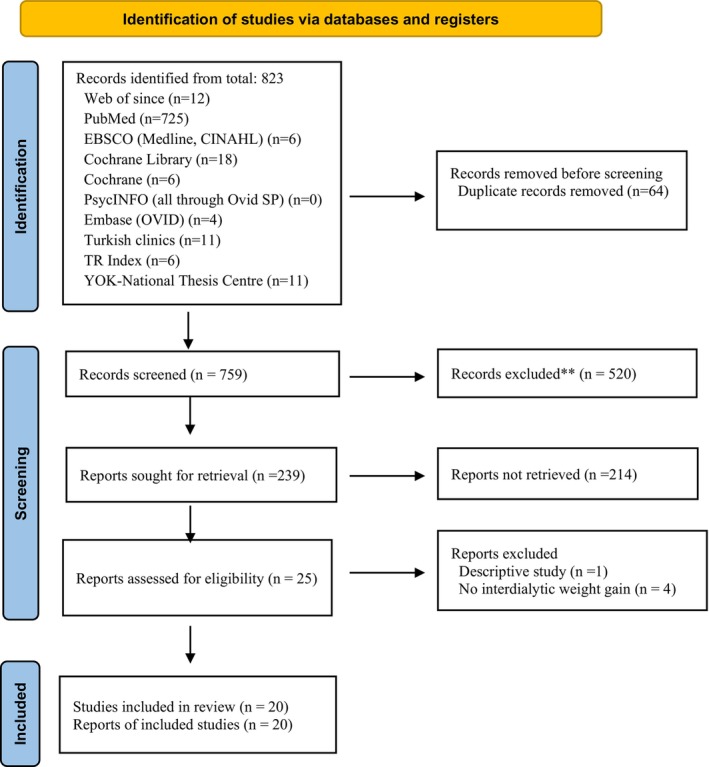
Prisma flow diagram (Page et al. 2021).

The previously stated search strategy yielded a total of 818 studies. After removing duplicates and screening titles and abstracts, studies were retained for full‐text evaluation (Figure 1). Finally, a total of 20 studies involving 1547 participants (intervention group: 660; control group: 627; single group: 260) were included in this systematic review and meta‐analysis. The studies included in the systematic review comprised eight randomised controlled experimental, nine quasi‐experimental with a control group, and three pre‐test‐post‐test controlled studies. The countries where the studies were conducted are Turkey, South Korea, the United States and India (Table 1).

**TABLE 1:** Characteristics of 20 included studies.

Authors (year)/country	Study design	Study year	Features of the intervention	Sample size	Average maternal age, years (SD)	Measuring tool/diagnostic test	Outcomes; main findings
Aliasgharpour et al. (2012)/Iran	Semi‐experimental	2010	The experimental group received a six‐session self‐efficacy promotion‐training program while the control group received the routine care. Mean body weight gain and self‐efficacy were measured before, immediately and 2 months after the study	Experimental: 32 Control: 31	Experimental 52.09 ± 11.31 Control 47.06 ± 15.84	– Demographic questionnaire – Self‐efficacy questionnaire (SUPPH)	Experimental group IDWG Before: 2.70 ± 0.7 Immediately: 2.20 ± 0.65 2 months after: 2.10 ± 0.67 Self‐efficacy Before: 67.3 ± 19.6 Immediately: 80.5 ± 16.9 2 months after: 86.9 ± 14.7 Control group IDWG Before: 2.60 ± 0.59 Immediately: 2.60 ± 0.60 2 months after: 2.80 ± 0.56 Self‐efficacy Before: 72.00 ± 20.5 Immediately: 70.70 ± 21.20 2 months after: 72.0 ± 20.50
An (2011)/Korea	Semi‐experimental	No information	The e‐mail group received an education program about the haemodialysis diet and drug administration by e‐mail twice a week for 6 weeks while the control group did not. (The e‐mail education program included material on the risks of fluid accumulation, sodium accumulation, and hyperkalemia, how to control fluid, sodium, and potassium, medication regimen, eating‐out, desirable menu, and so on)	Experimental: 19 Control: 21	Experimental 62.59 ± 2.06 Control 63.70 ± 2.42	– Demographic questionnaire – Laboratory and IWG parameters were used to measure	Experimental group IDWG: 2.17 ± 0.82 Control group IDWG: 2.84 ± 0.94
Başer and Mollaoğlu (2019)/Turkey	Semi‐experimental	2017	The content and implementation of the education program (the training booklet was given to the patients)	Experimental: 38 Control: 40	Experimental 35–50 age: 5 51–65 age: 22 65 and upper age: 11 Control 35–50 age: 4 51–65 age: 21 65 and upper age: 15	– Demographic questionnaire – Dialysis diet and fluid non‐adherence questionnaire (DDFQ) – Haemodialysis Patients Fluid Control Scale (HPFCS)	Experimental group IDWG: 1.89 ± 1.06 Systolic BP mmHg: 121.05 ± 12.47 Diastolic BP mmHg: 71.12 ± 6.36 Control group IDWG: 2.26 ± 1.96 Systolic BP mmHg: 123.87 ± 12.13 Diastolic BP mmHg: 76.12 ± 7.26
Bulantekin Düzalan (2014)/Turkey	Semi‐experimental	2014	About 60 min of individual face‐to‐face training on nutrition, the importance of protein‐phosphorus and salt‐restricted diet and fluid restriction in CKD, HD and CKD, and then the training material prepared by the researcher was given. A post‐test was administered 1 month after the training	Experimental: 40 Control: 40	Experimental 48.83 ± 11.75 Control 56.23 ± 11.21	– Demographic questionnaire – Haemodialysis Patients' Dietary Knowledge Scale (AIDS) – Attitude Scale (AHHRS) – Behaviour Scale (ADSS)	Experimental group Weight before dialysis: 72.76 ± 13.49 Weight after dialysis: 70.36 ± 13.30 Systolic BP mmHg: 117.50 ± 30.19 Diastolic BP mmHg: 71.00 ± 13.92 Control group Weight before dialysis: 67.26 ± 12.59 Weight after dialysis: 64.30 ± 12.00 Systolic BP mmHg: 107.00 ± 17.28 Diastolic BP mmHg: 70.00 ± 10.38
Chang et al. (2021)/South Korea	Semi‐experimental	2019	Measurements were taken at baseline, immediately after the 6‐week intervention, and 4 months post‐intervention. The experimental and comparison groups attended the 60‐min fluid‐adherence program once a week for 6 weeks. The experimental group received additional auricular acupressure (AA) at three auricular acupoints for 6 weeks	Experimental group (AA + fluid‐restriction adherence program): 29 Comparison group (fluid‐restriction adherence program): 27 Control group (usual care): 28	Experimental 67.4 ± 11.4 Comparison 61.8 ± 14.1 Control 63.2 ± 14.9	– Fluid Control in Haemodialysis Patient Scale – Diet‐related quality of life – Salivary flow rate	Experimental group IDWG Week 6: 1.60 ± 0.59 Week 22: 1.50 ± 0.87 Comparison group IDWG Week 6: 1.64 ± 0.75 Week 22: 1.73 ± 0.74 Control group IDWG Week 6: 1.94 ± 0.82 Week 22: 1.99 ± 0.85
Cho and Park (2020)/Korea	Semi‐experimental	2017	A 70‐min video program was used, which included a self‐performance management program, which took 2 weeks to complete. By recording the training contents on the tablet PC, the patients were allowed to manage themselves, they repeated the 2‐week course twice, and it was completed in a total of 6 weeks. Brochure was used for the control group	Experimental: 23 Control: 23	Experimental 55.00 ± 9.82 Control 53.66 ± 10.50	– Demographic and disease‐related characteristics of the participants – Self‐care knowledge – Self‐care behaviour	Experimental group IDWG: 1.77 ± 0.46 Control group IDWG: 1.90 ± 0.34
Cakır (2013)/Turkey	Pre post intervention	2010	First, all questionnaires were administered, and in the 1st month, ‘Medical Nutrition Therapy’ training was given. Relatives of the patients who could be reached were given “Medical Nutrition Therapy” training, and in the 3rd month, the patients were trained again in the clinic and their relatives by telephone. The last test was done at 6 months	Experimental: 60	18–29 age: %1.4 30–39 age: %6.8 40–49 age: %1.9 50–59 age: %20.6 60–69 age: %30.1 70–79 age: %19.2	– Patient Survey Form – Nutrition information status evaluation form – Nutrition monitoring criteria form – Subjective global evaluation form – SF‐36 Quality of Life Scale	Pre‐test IDWG: 2.98 ± 1.20 Post‐test IDWG: 3.73 ± 0.91
Cayır Yılmaz (2017)/Turkey	Semi‐experimental	2017	The experimental group was given pre‐test application and training booklets prepared based on RAM. In the following period, training practices were carried out for four main topics created according to RAM for 2 months, once every 14 days, and the training was repeated over the phone in the 4th, 5th and 6th months	Experimental: 42 Control: 39	Experimental 18–30 age: 7 31–45 age: 10 46–64 age: 17 65 and upper age: 8 Control 18–30 age: 5 31–45 age: 6 46–64 age: 16 65 and upper age: 12	– Haemodialysis patient information form – Illness Acceptance Scale (IDS) – Haemodialysis Patients Fluid Control Scale (HPFCS)	Experimental group IDWG: 2.60 ± 1.06 Systolic BP mmHg:125.48 ± 23.91 Diastolic BP mmHg:76.19 ± 19.25 Weight before dialysis: 69.38 ± 18.43 Weight after dialysis: 66.88 ± 18.14 HPFCS score: 55.31 ± 6.41 Control group IDWG: 2.97 ± 1.44 Systolic BP mmHg:136.15 ± 26.62 Diastolic BP mmHg:77.44 ± 11.86 Weight before dialysis: 68.47 ± 14.43 Weight after dialysis: 65.73 ± 13.94 HPFCS score: 50.67 ± 6.23
Jafari et al. (2014)/Iran	Pre post intervention	2011	Diet education, including face to face training with instruction booklets, were conducted in the two sessions. Having carried out the educational program, blood pressure and interdialytic weight gain were measured and recorded 1 month before and during three stages and after the educational program by researcher‐designed checklists	Experimental:100	56.2 ± 15.14	The instrument used for data collection was a two‐part researcher‐designed checklist which consisted of questions on demographic information, knowledge of consumption of food, systolic and diastolic hypertension control, as well as the weight control of the patients	Two weeks after training Weight before haemodialysis:64/40 ± 14/07 Systolic BP mmHg: 134/0 ± 17/70 Diastolic BP mmHg:79/03 ± 11/05 At completion of the second month Weight before haemodialysis:64 ± 5/14 Systolic BP mmHg: 128/2 ± 13/08 Diastolic BP mmHg: 79/02 ± 7/5
Kacaroglu Vicdan and Gulseven (2016)/Turkey	Randomised controlled trial	2012	In Nursing Initiative, a training manual prepared through RAM, literature review and expert opinions were used as the material. The training manual is composed of four sections according to RAM. All trainings were provided by the same researcher during haemodialysis sessions. They were repeated at certain intervals	Experimental: 41 Control: 41	Experimental: 64.12 ± 12.55 Control: 62.27 ± 9.76	– Haemodialysis patient evaluation form – Functional performance inventory short form – The coopersmith self‐esteem Inventory	Experimental group Weight before haemodialysis: 70.12 ± 15.2 Systolic BP mmHg: 119.7 ± 13.6 Diastolic BP mmHg:72.4 ± 7.6 Control group Weight before haemodialysis:71.23 ± 13.4 Systolic BP mmHg:125 ± 17.1 Diastolic BP mmHg:76.3 ± 8.5
Karabey (2017)/Turkey	Randomised controlled trial	2016	Training on the Follow‐up of Obtained and Extracted (MCT) was given and a record was requested in the notebooks. Then, in line with the training booklet prepared based on the literature, 45 min were given to the patients in the intervention group. Individual fluid management training was provided	Experimental: 40 Control: 40	40–60	– Demographic questionnaire – The Fluid Control Scale – The Short Form‐36 Quality of Life Scale	Experimental group IDWG First Interview:1.640 ± 480 Second Interview:1.450 ± 0.350 Third Interview:1.080 ± 0.720 Control group IDWG First Interview: 2.150 ± 730 Second Interview: 2.150 ± 730 Third Interview: 2.230 ± 720
Min and Park (2020)/South Korea	Semi‐experimental	2018	To enhance app utilisation, we provided the participants with 1 week of education on how to use their smartphones. Re‐education was conducted 4 weeks later. Both the experimental and control groups underwent regular blood tests and health check‐ups by a physician and were provided with education on diet and interim blood tests	Experimental: 28 Control: 28	Experimental: 61.96 ± 11.42 Control: 62.39 ± 14.48	– Basic psychological needs – Self‐efficacy – General characteristics (Sex, age, comorbidity, living arrangement, duration of haemodialysis, job, education, monthly income) – Blood test	Experimental group IDWG: 3.91 ± 1.72 Self‐efficacy: 31.35 ± 3.65 Control group IDWG: 3.65 ± 1.34 Self‐efficacy: 21.89 ± 3.99
Moattari et al. (2012)/Iran	Randomised controlled trial	2010	Patients in the experimental group completed a 6‐week empowerment program that consisted of four individual and two group counselling sessions. Six weeks after intervention, post‐test data were obtained from both groups in the same manner as the pre‐test. To run group counselling, the intervention group was divided into two smaller groups, each attending two sessions for 1.5–2 h	Experimental: 25 Control: 23	Experimental: 38.56 ± 11.4 Control: 37.3 ± 12.79	– Self‐care self‐efficacy – Quality of Life Score questionnaire	Experimental group IDWG:2.08 ± 0.71 Systolic BP mmHg: 129.02 ± 11.58 Diastolic BP mmHg: 77.48 ± 6.08 Self‐efficacy: 97.80 ± 11.15 Quality of Life Score: 20.47 ± 2.50 Control group IDWG:2.52 ± 0.70 Systolic BP mmHg:143.37 ± 11.56 Diastolic BP mmHg: 83.10 ± 6.08 Self‐efficacy: 85.78 ± 11.16 Quality of Life Score:17.54 ± 2.51
Ozdemir (2021)/Turkey	Randomised controlled trial	2020	Education booklet: It is based on the Roy Adaptation Model and developed by the researcher. Data were collected within 2 h of the first session, then 30–45 min of training was given to the experimental group. After 1 month, the training was repeated, and after 3 months the final test was performed	Experimental: 53 Control: 54	Experimental: 61.96 ± 11.42 Control: 62.39 ± 14.48	– Demographic questionnaire – Fluid Control Scale in Haemodialysis Patients – Dialysis Symptom Index – Nottingham health profile	Experimental group 1. month IDWG: 2.83 ± 0.71 Systolic BP mmHg:126.79 ± 17.92 Diastolic BP mmHg:74.06 ± 9.46 Weight before dialysis: 73.52 ± 12.81 Weight after dialysis: 70.18 ± 13.71 3. month IDWG: 3.11 ± 0.93 Systolic BP mmHg:127.74 ± 18.46 Diastolic BP mmHg: 73.21 ± 9.15 Weight before dialysis: 73.53 ± 13.30 Weight after dialysis: 70.55 ± 12.88 Control group 1. month IDWG: 3.27 ± 0.92 Systolic BP mmHg:130.65 ± 22.40 Diastolic BP mmHg: 74.63 ± 9.85 Weight before dialysis: 72.79 ± 16.69 Weight after dialysis: 68.73 ± 15.53 3. month IDWG: 3.32 ± 0.87 Systolic BP mmHg:133.43 ± 21.58 Diastolic BP mmHg: 77.87 ± 9.89 Weight before dialysis: 71.71 ± 15.73 Weight after dialysis: 68.56 ± 15.53
Park and Kim (2019)/South Korea	Semi‐experimental	2016	In this study, the self‐management program consisted of mobile application usage, SMS messages twice a week, and face‐to‐face counselling and training twice a month. The mobile application included self‐awareness, knowledge and skill acquisition, self‐efficacy development and active participation. It concerned questions about HD self‐management (e.g., seasonal fruit selection, travel precautions, relationships between medications and food, and how to take medications)	Experimental: 42 Control: 42	Experimental: 51.48 ± 10.15 Control: 48.93 ± 9.37	The general characteristics, self‐efficacy, and treatment compliance were investigated through questionnaires.	Experimental group IDWG: 4.43 ± 1.41 Self‐efficacy: 36.31 ± 3.50 Control group IDWG: 4.91 ± 1.42 Self‐efficacy: 28.24 ± 4.76
Park and Kim (2022)/South Korea	Semi‐experimental	2020	The intervention group (*n* = 20) received a health literacy‐based self‐management intervention for patient–family caregiver dyads, whereas the control group (*n* = 23) received standard informational messages through a short messaging service for 8 weeks. All participants were assessed for haemodialysis knowledge, self‐efficacy, family support, self‐management, the ratio of interdialytic weight gain to dry weight, and serum phosphorus and potassium levels at pretest and posttest	Experimental: 20 Control: 23	Experimental: 60.40 (±13.06) Control: 60.35 (±9.62)	– Haemodialysis Knowledge Scale – Haemodialysis Self‐Efficacy Scale – The Family Support Scale – The haemodialysis self‐management instrument	Experimental group IDWG: 3.83 ± 1.24 Self‐efficacy: 28.80 ± 5.86 Control group IDWG: 3.94 ± 0.92 Self‐efficacy: 30.87 ± 4.50
Sevick et al. (2016)/The United States	Randomised controlled trial	2012	Both the intervention and control groups received face‐to‐face training on the HD diet for 16 weeks. The intervention group also received behavioural counselling based on social cognitive theory and monitored their diet daily using computers	Experimental: 93 Control: 86	Experimental: 62 (53–71) Control: 60 (59–69)	Laboratory and IWG parameters were used to measure treatment adherence. For Baseline, Week 8, Week 12 and Week 16	Experimental group Week 8 IDWG: 1.1 Week 12 IDWG: 1.2 Week 16 IDWG:1.1 Control group Week 8 IDWG: 1.2 Week 12 IDWG: 1.2 Week 16 IDWG: 1.1
Kurt et al. (2012)/Turkey	Pre post intervention	No information	Salt and fluid restriction training was given once a week for 2 weeks. In the first week, colour‐visual and written training, and in the second week only in written form, the patients were given training by a doctor with the materials that were given to them for further examination	Experimental: 100	56.2 ± 13.5	(Pre‐posttest)	Pre‐Test IDWG: 2.72 ± 0.6 Systolic BP mmHg: 134.5 ± 13.6 Diastolic BP mmHg: 79.8 ± 4.5 Post‐test IDWG: 2.58 ± 0.7 Systolic BP mmHg: 132.9 ± 12.5 Diastolic BP mmHg: 79.9 ± 4.07
Valsaraj et al. (2021)/India	Randomised controlled trial	2014	Cognitive behavioural therapy (CBT) training was given and included the use of fluids, diet, and medication	Experimental: 33 Control: 34	20–65	– Hospital Anxiety Depression Scale – Individuals recorded information – Haemodialysis adherence scale	Experimental group 3rd month follow‐up results IDWG: 3.18 ± 0.77 Systolic BP mmHg: 144.85 ± 13.49 Diastolic BP mmHg: 87.27 ± 6.26 Control group IDWG:4.30 ± 0.57 Systolic BP mmHg: 162.65 ± 17.81 Diastolic BP mmHg: 94.12 ± 7.83 Experimental group 6th month follow‐up results IDWG: 3.24 ± 0.63 Systolic BP mmHg: 145.15 ± 12.78 Diastolic BP mmHg: 88.18 ± 6.35 Control group IDWG: 4.69 ± 0.47 Systolic BP mmHg:165.59 ± 17.09 Diastolic BP mmHg: 95.88 ± 7.83
Zhianfar et al. (2020)/Iran	Randomised controlled trial	2018	Sociodemographic and clinical data were collected at baseline in both groups and the proposed intervention was tailored to the individual participants' literacy level at two layers, that is, patients' and their families and nursing staff over 8 weeks' time	Experimental: 35 Control: 35	Experimental < 40: %5.7 40–60: %48.6 > 60: %45.7 Control < 40: %14.3 40–60: %45.7 > 60: %40	– The beck depression inventory (BDI‐SF) – The Multidimensional Scale of Perceived Social Support (MSPSS) – The patient satisfaction with nursing care quality questionnaire (PSNCQQ) – The end‐stage renal disease adherence questionnaire (ESRD‐AQ) – The World Health Organisation quality of life (WHOQOL‐SF) questionnaire	Experimental group 1. month IDWG: 2518.18 Quality of Life Score: 91.24 ± 13.38 3. month IDWG: 2703.03 Quality of Life Score:89.67 ± 14.33 Control group 1. month IDWG: 3012.12 Quality of Life Score: 80.48 ± 21.03 3. month IDWG: 3069.70 Quality of Life Score: 76.67 ± 21.69

### 3.2 Evaluation of Publication Quality

**FIGURE 2:**
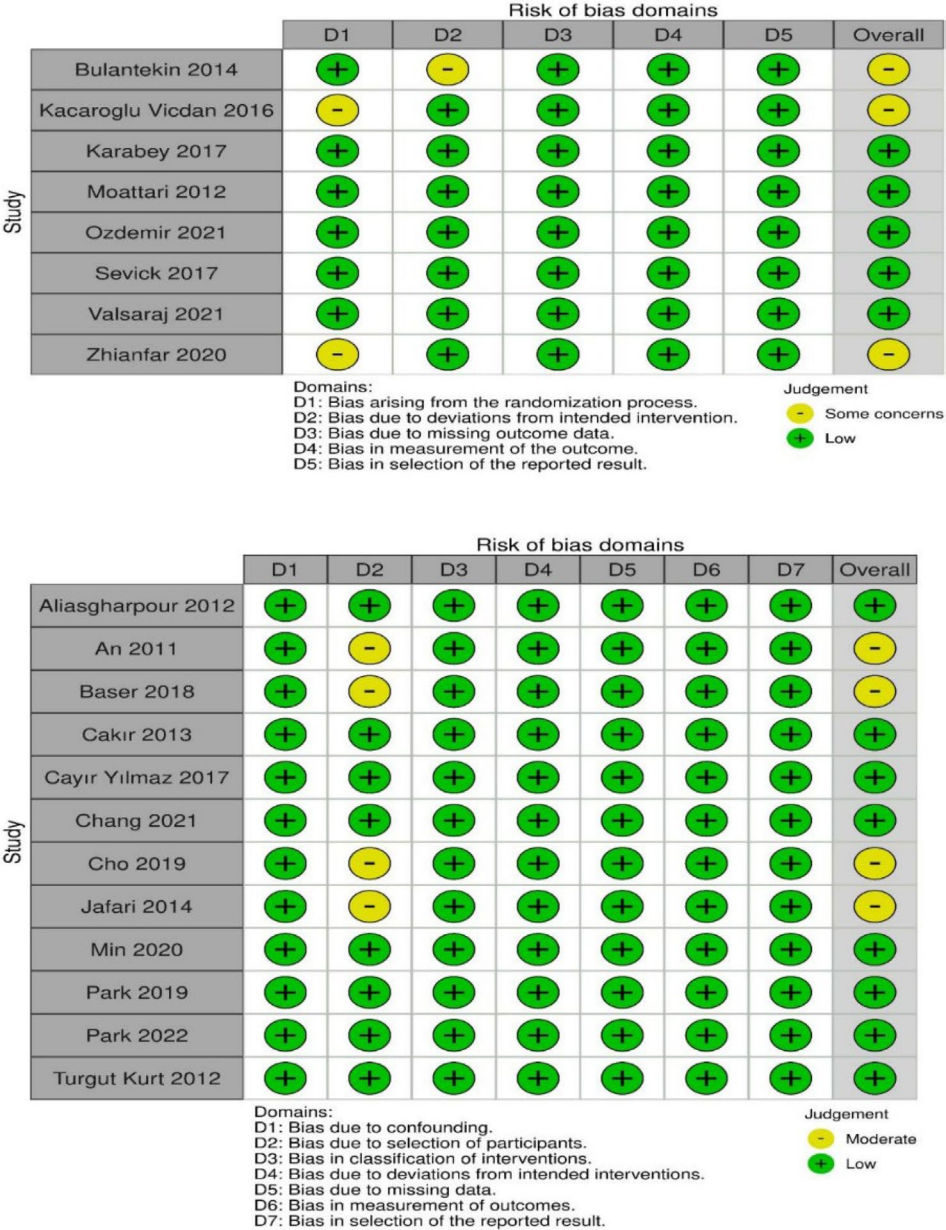
Risk of bias summary of Cochrane quality assessment for included studies.

Among the randomised controlled experimental studies included in the review, five studies were assessed to have a low risk of bias. Three studies were classified as having some concerns regarding bias. Specifically, two studies had some concerns related to the randomisation process, and one study had some concerns regarding deviation from the intervention. For the non‐randomised studies, eight studies were determined to have a low risk of bias, while four studies were classified as having a moderate risk of bias. The studies with a moderate risk of bias were found to have biases primarily related to participant selection (Figure 2).

### 3.3 Meta‐Analysis Results

#### 3.3.1 Meta‐Analysis Results on IDWG

**FIGURE 3:**
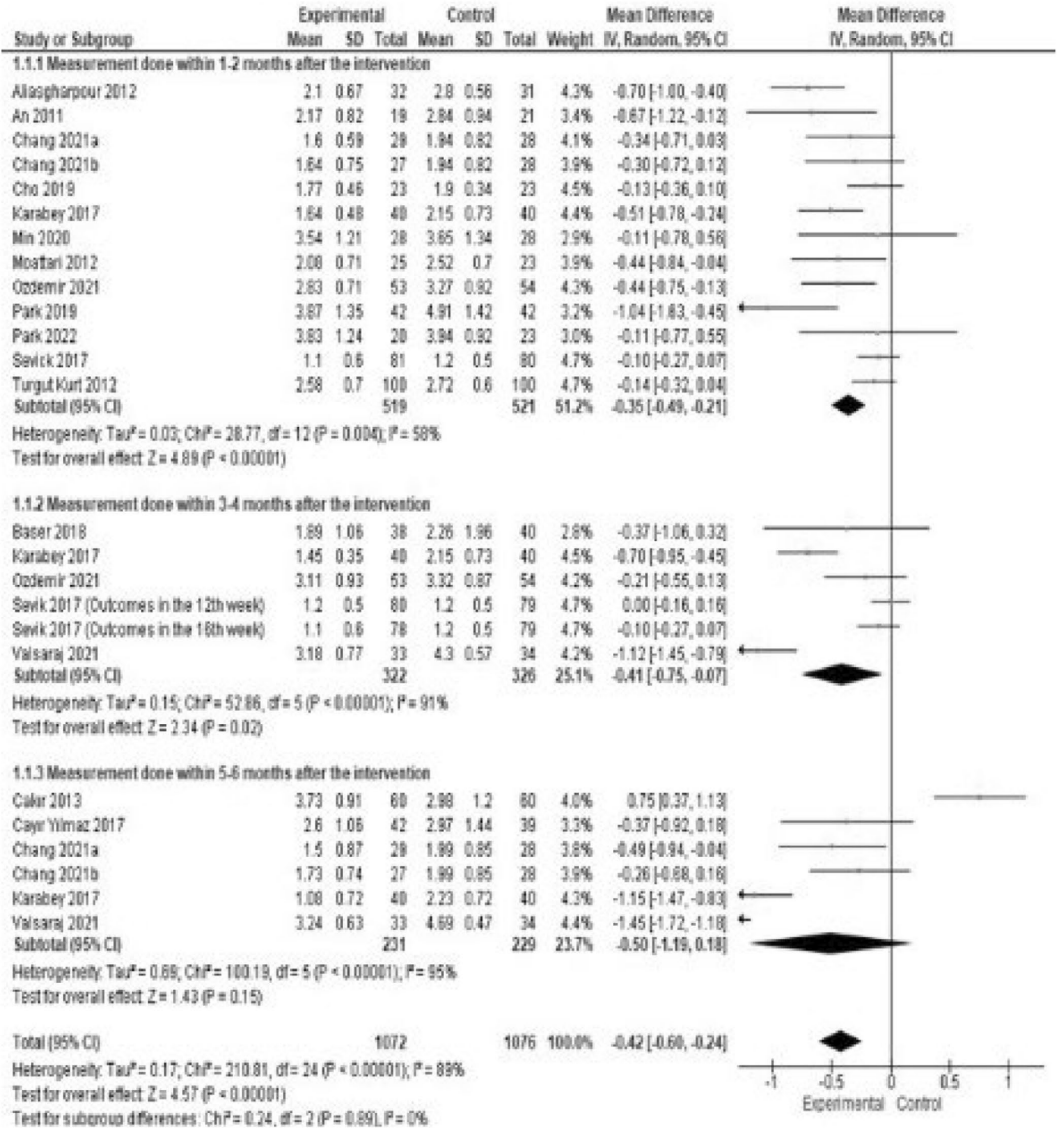
Subgroup analysis by time of data collection of intradialytic weight gain.

The combined results of 16 studies reporting the effects of nutrition education on the change in IDWG in HD patients showed a statistically significant difference between the experimental and control groups (MD: −0.42 kg/m^2^, 95% CI: −0.60 to −0.24, *Z* = 4.57, *p* < 0.01). Subgroup analysis conducted based on the data collection time revealed that this significant effect persisted in measurements conducted at 1–2 months and 3–4 months, but was not effective at 5–6 months (MD: −0.35, *Z* = 4.89, *p* < 0.01; MD: −0.41, *Z* = 2.34, *p* = 0.02; MD: −0.50, *Z* = 1.43, *p* = 0.15, respectively; Figure 3). Furthermore, the meta‐regression analysis conducted with the moderator variable of data collection time was also found to be statistically significant (*Q* = 6.58, df = 2, *p* = 0.03).

Additionally, the meta‐regression analysis conducted with the moderator variable of country in this study was found to be statistically significant (*Q* = 32.32, df = 4, *p* < 0.01). In the subgroup analysis conducted to determine which country contributed to this difference, it was determined that the significant effect persisted for countries other than the United States.

The meta‐regression analyses conducted with the moderator variables of study design, training programme implementation, and risk of bias on IDWG data revealed that the results were not statistically significant (respectively: *Q* = 2.92, df = 1, *p* = 0.08; *Q* = 2.38, df = 2, *p* = 0.30; *Q* = 0.42, df = 1, *p* = 0.52).

#### 3.3.2 Meta‐Analysis Results on Haemodialysis Fluid Control Scale Score

The scale, which determines the knowledge, behaviour, and attitudes of HD patients about fluid restriction, consists of a total of 24 items and three sub‐dimensions. The lowest score obtained from the scale is 24 and the highest score is 72, and an increase in the score indicates that patients' compliance with fluid control increases.

**FIGURE 4:**
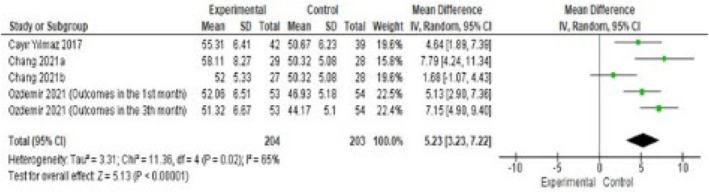
Haemodialysis fluid control scale by meta‐analysis.

According to the combined meta‐analysis results of studies included in this research that used the ‘Hemodialysis Fluid Control Scale’ to determine the effect of education provided to HD patients, it was found that the provided education was effective in reducing IDWG (MD: 5.23, 95% CI: 3.23–7.22, Z = 5.13, *p* < 0.01; Figure 4).

#### 3.3.3 Meta‐Analysis Results on Systolic and Diastolic Blood Pressure

**FIGURE 5:**
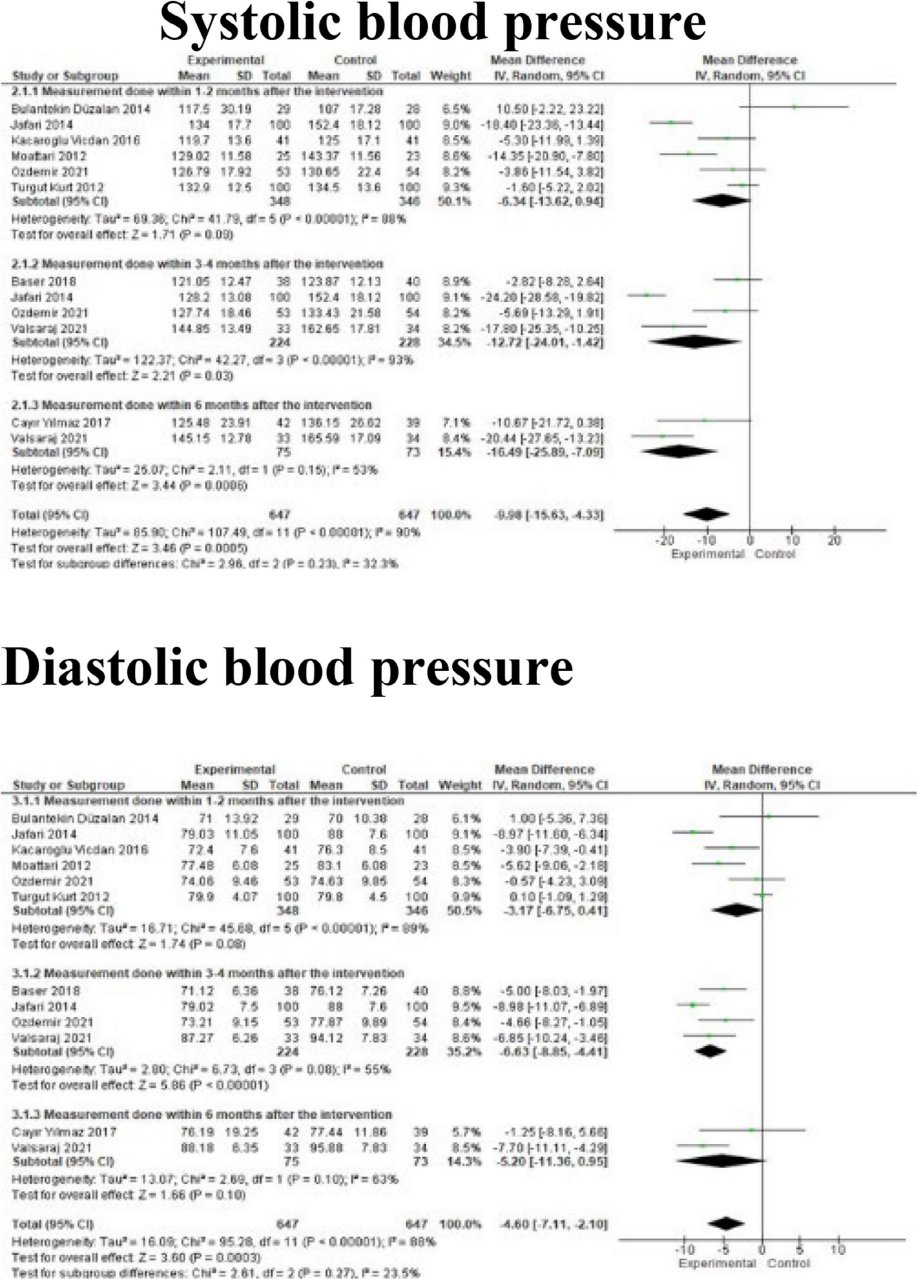
Meta‐analysis findings on systolic and diastolic blood pressure.

The combined results of nine studies included in the meta‐analysis showed that nutrition education had a statistically significant effect in reducing systolic and diastolic blood pressure (respectively; MD: −9.98 mmHg, 95% CI: −15.63–4.33, *Z* = 3.46, *p* = 0.01; MD: −4.60 mmHg, 95% CI: −7.11 −2.10, *Z* = 3.60, *p* = 0.01; Figure 5).

In subgroup analysis based on the evaluation time of systolic blood pressure, it was found that this education was not effective in the 1–2 month period, but it was effective in the 3–4 month and 5–6 month periods (respectively; MD: −6.34, *Z* = 1.71, *p* = 0.09; MD: −12.72, *Z* = 2.21, *p* = 0.030; MD: −16.49, *Z* = 3.44, *p* = 0.01). Similarly, in the subgroup analysis based on the evaluation time of diastolic blood pressure, it was determined that nutrition education was not effective in reducing diastolic blood pressure in the 1–2 month and 5–6 month periods, but it was effective in the 3–4 month period (respectively; MD: −3.17, *Z* = 1.74, *p* = 0.08; MD: −5.20, *Z* = 1.66, *p* = 0.10; MD: −6.63, *Z* = 5.86, *p* < 0.01).

#### 3.3.4 Meta‐Analysis Results on Self‐Efficacy and Quality of Life

**FIGURE 6:**
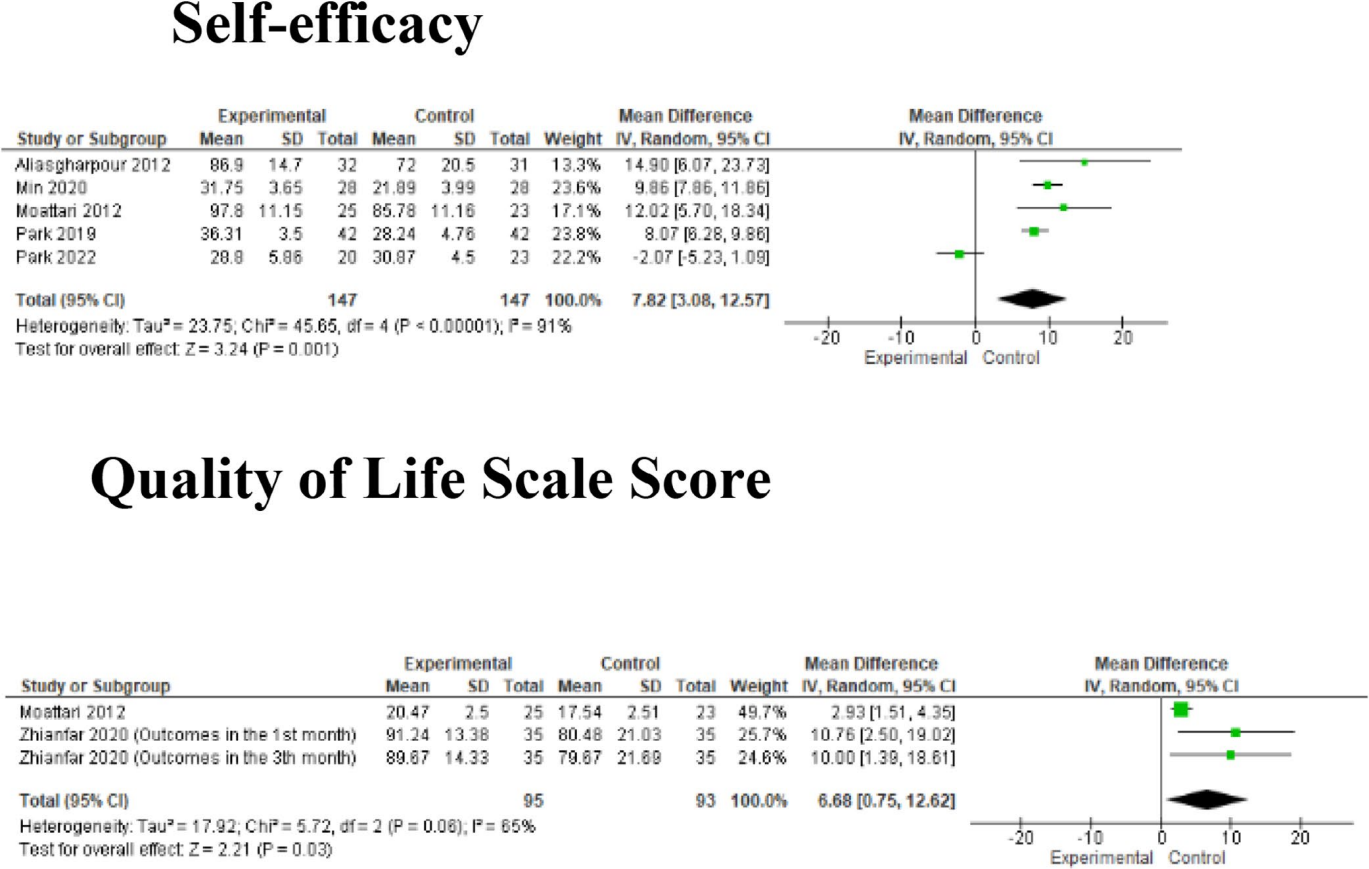
Meta‐analysis findings on self‐efficacy and quality of life.

Five studies were included in the meta‐analysis regarding the effect of nutrition education on self‐efficacy, and two studies reported the effect on quality of life. The combined results of these studies revealed that there was a statistically significant difference between the groups (respectively; MD: 7.82, 95% CI: 3.08–12.57, *Z* = 3.24, *p* = 0.001; MD: 6.68, 95% CI: 0.75–12.62, *Z* = 2.21, *p* = 0.03; Figure 6).

### 3.4 Publication Bias

**FIGURE 7:**
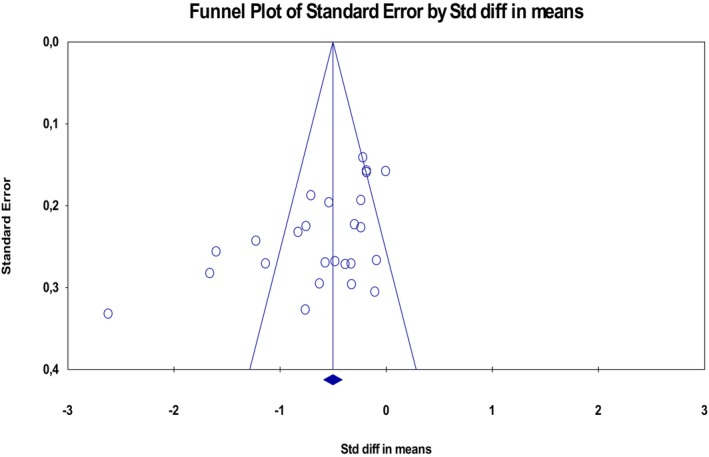
Publication bias related to intradialytic weight gain data funnel plot.

In the evaluation conducted with the IDWA dataset in this study, it was determined that there was a statistically significant publication bias among the studies (*t* = 3.21, df = 23, *p* = 0.01; Figure 7).

## 4 Discussion

### 4.1 Importance of Evidence Summary for the Prevention and Management of Intradialytic Weight Gain in Haemodialysis Patients

This meta‐analysis, conducted to investigate the impact of nutrition education on IDWG in haemodialysis patients, demonstrates that the provided education has a statistically significant effect in reducing IDWG. The findings of this study are valuable in providing comprehensive and up‐to‐date information on the subject. Haemodialysis patients often face difficulties in adhering to fluid intake during their treatments, which leads to the emergence of many complications, as well as negative effects on mortality and morbidity rates (Goto et al. 2021; Oller et al. 2018; Liu et al. 2020).

Previous studies have shown that HD patients often experience inadequate adherence to IDWG guidelines (Günalay et al. 2017; Karabulutlu and Yılmaz 2019; Yangöz and Ozer 2020). In this study, it was found that nutrition education provided to HD patients was effective in reducing IDWG, and this significant effect varied according to the measurement time and the country where the research was conducted. Baraz et al. (2010) found that nutrition education provided to HD patients was effective in improving nutrition and fluid adherence, with an increase in IDWG adherence in 76% of all participants. Similarly, a randomised controlled study showed a moderate effect in reducing IDWG and improving fluid intake adherence in HD patients (Howren et al. 2016). In a study conducted by Isnaini et al. (2021) with HD patients, it was shown that providing education in plain language had a significant impact on increasing patients' self‐efficacy and knowledge levels but was not effective in reducing IDWG.

In this meta‐analysis, a reverse relationship was observed between patients' fluid intake adherence and the duration of education. Subgroup analysis based on data collection time showed that the significant effect of education continued immediately after the intervention and shortly thereafter but decreased over time. Murali et al. (2019) conducted a systematic review and meta‐analysis showing that most interventions had partial and short‐term effects on improving diet, fluid intake and medication adherence strategies moderately. However, in a study by Hanifi et al. (2019) investigating the effects of consultations and follow‐up phone calls on biochemical markers and IDWG in HD patients, no statistically significant improvements were observed in IDWG and biochemical values with the education provided.

In this study, it was found that nutrition education provided to HD patients has a positive effect on increasing the HD Fluid Control Scale score. Studies conducted with HD patients have indicated that as the HD Fluid Control Scale average score increases, IDWG and UF volume decrease (Balım and Pakyüz 2016; Yangöz and Ozer 2020). Biçer and Yılmaz Karabulutlu (2020) reported that higher IDWG is associated with decreased overall compliance with fluid restriction and negative behaviours and attitudes toward IDWG, while patients with higher fluid control levels exhibit lower IDWG. In a randomised controlled study conducted with HD patients, no statistically significant difference was found in the mean scores of the HD Fluid Control Scale subscales and total score in the initial measurement. However, in the subsequent measurement of the same study, statistically significant differences were observed in the total score and behaviour and attitude subscale scores (Yangöz and Ozer 2020).

The findings of this meta‐analysis indicate that nutrition education has a statistically significant effect in reducing both systolic and diastolic blood pressure. Kurt et al. (2012) conducted a study showing that education provided to HD patients significantly reduced IDWG and systolic blood pressure but did not have a significant effect on reducing diastolic blood pressure. Similarly, Koşar Şahin et al. (2018) observed a significant decrease in IDWG, UF volume, and pre‐dialysis systolic and diastolic blood pressures with increased adherence to fluid intake in HD patients. These findings demonstrate the importance of controlling high fluid adherence in HD patients for managing IDWG and reducing hypertension.

The study determined that nutrition education is effective in improving self‐efficacy and quality of life. In a quasi‐experimental study investigating the impact of health education delivered through video on the self‐efficacy of HD patients, Ren et al. (2022) found that patients in the intervention group exhibited greater improvement in average self‐efficacy scores compared to the control group. However, no significant differences were observed in between‐group and within‐group comparisons. Welch et al. (2013) examined the effect of a mobile application on diet and fluid intake in HD patients and reported no significant differences in terms of self‐efficacy between groups or over time. Biçer and Yılmaz Karabulutlu (2020) found that patients with higher levels of self‐efficacy demonstrated better adherence to IDWG. Several similar studies have shown that educational programmes provided to HD patients enhance their self‐efficacy, reduce IDWG, and positively contribute to improving their quality of life (Pack and Lee 2021; Ramezani et al. 2019; Welch et al. 2013; Isnaini et al. 2021). Education aimed at improving patients' treatment and fluid intake adherence enhances their knowledge and awareness, which in turn increases their self‐efficacy, helps prevent potential complications, and improves treatment effectiveness.

### 4.2 Implications for Nursing and Health Policy

Education provided to HD patients under the leadership of nurses is a beneficial intervention for reducing IDWG in HD patients, and study results have demonstrated the effectiveness of this approach. Additionally, this study has revealed that the education provided in this intervention is effective in reducing patients' systolic and diastolic blood pressure, improving laboratory results, enhancing self‐efficacy, and increasing their quality of life. The findings of this review provide robust evidence for the implementation of regular education by nurses in clinical practice to ensure effective treatment in HD patients. However, further research is needed to validate these findings.

The management of HD continues to pose significant challenges. High IDWG in HD patients leads to hypertension, left ventricular hypertrophy, pulmonary oedema and congestive heart failure, all of which contribute to high mortality and morbidity rates (Goto et al. 2021; Oller et al. 2018; Liu et al. 2020). Therefore, ensuring adherence to the recommended diet programme to reduce these problems, improve quality of life, and lower mortality rates is crucial (Kulaksız 2019; Naseri‐Salahshour et al. 2020; Beerendrakumar et al. 2018). Increasing adherence among HD patients can be achieved through regular patient education interventions led by nurses. As a result, future studies should provide robust guidelines to develop feasible and sustainable interventions for reducing IDWG in HD patients, focusing on nurse‐supported care planning and regular education.

## 5 Conclusions

In conclusion, this meta‐analysis provides strong evidence that nutritional education effectively reduces IDWG in HD patients, particularly in the short term. Additionally, it has been observed that achieving fluid adherence in HD patients leads to a decrease in high blood pressure and an enhancement in the quality of life. These findings are in line with previous literature on the subject. The significant reduction in IDWG observed in different countries regardless of geographical location emphasises the effectiveness of educational interventions. Furthermore, this study found that the effectiveness of interventions remained consistent across different study designs, implementation methods, and levels of bias risk.

Based on the results of this meta‐analysis, it is recommended that regular patient education sessions focusing on fluid adherence and reducing IDWG should take precedence to prevent complications associated with high fluid intake, and enhance overall quality of life in HD patients. Healthcare providers, administrators, and educators should consider integrating these research findings into healthcare policies and practices.

Future research should prioritise conducting similar studies that further support the effectiveness of educational interventions for HD patients. Experimental studies investigating innovative approaches to reduce and manage IDWG should be encouraged. Healthcare providers can improve outcomes and enhance the well‐being of HD patients through the implementation of evidence‐based educational programmes and strategies.

## 6 Strengths and Limitations

The systematic review and meta‐analysis conducted in this study possess several strengths. First, an extensive search strategy was employed to identify relevant sources, ensuring comprehensive coverage of the literature. Additionally, the inclusion of studies conducted in different countries enhances the generalisability of the findings. The currency and high quality assessment scores of the included studies further contribute to the strength of the study. Moreover, the large combined sample size of 1547 participants enhances the statistical power and precision of the analyses. The utilisation of various analyses, including meta‐regression, also adds robustness to the findings. However, it is important to acknowledge certain limitations that may affect the strength of the study. One notable limitation is the high heterogeneity observed among the included studies in all conducted meta‐analyses. This heterogeneity could be attributed to variations in study design, population characteristics, intervention protocols and outcome measures across the included studies. To address this limitation, the Random Effects model was employed for the meta‐analyses, which considers the presence of heterogeneity and provides more conservative estimates.

## Author Contributions

**Sevda Tüzün Özdemir:** conceptualization, methodology, software, validation, formal analysis, investigation, resources, data curation, writing, original draft, writing, review and editing, visualisation. **Zekiye Karaçam:** conceptualization, methodology, software, validation, formal analysis, investigation, resources, data curation, writing, original draft, writing, review and editing, visualisation, supervision. **Öznur Usta Yeşilbalkan:** conceptualization, resources, review and editing, visualisation.

## Ethics Statement

The authors have nothing to report.

## Conflicts of Interest

The authors declare no conflicts of interest.

## Data Availability

The dataset that supports the findings in this study is available from the corresponding author upon reasonable request.
